# Supplementary material for: SUMOylation of Rho-associated protein kinase 2 induces goblet cell metaplasia in allergic airways
Source: Nat Commun. 2023 Jul 1;14:3887. doi: 10.1038/s41467-023-39600-4 (PMC10314948; doi:10.1038/s41467-023-39600-4)
Supplement: Supplementary file 1 — Supplementary Information [file 41467_2023_39600_MOESM1_ESM.pdf]

SUMOylation of Rho-associated protein kinase 2 induces goblet cell metaplasia in allergic airways

Dan Tan[1,2,*], Meiping Lu[3,*,†], Yuqing Cai[3], Weibo Qi[4], Fugen Wu[5], Hangyang Bao[1], Meiyu Qv[1], Qiangqiang He[1], Yana Xu[1], Xiangzhi Wang[3], Tingyu Shen[1], Jiahao Luo[1], Yangxun He[1], Junsong Wu[6], Lanfang Tang[3], Muhammad Qasim Barkat[1], Chengyun Xu[1,2,3,†], Ximei Wu[1,2,†]

[1]Department of Pharmacology, Zhejiang University School of Medicine, Hangzhou 310058, China;

[2]Key Laboratory of CFDA for Respiratory Drug Research, Zhejiang University School of Medicine, Hangzhou 310058, China;

[3]National Clinical Research Center for Child Health, the Children's Hospital of Zhejiang University School of Medicine, Hangzhou 310053, China;

[4]Department of Thoracic Surgery, the Affiliated Hospital of Jiaxing University, Jiaxing 314001, China;

[5]Department of Paediatrics, the First People's Hospital of Wenling City, Wenling City 317500, China;

[6]Department of Critical Care Medicine, the First Affiliated Hospital, Zhejiang University School of Medicine, Hangzhou 310003, China.

Running title: SUMOylation of ROCK2 in asthma

*These authors contribute equally to this work.

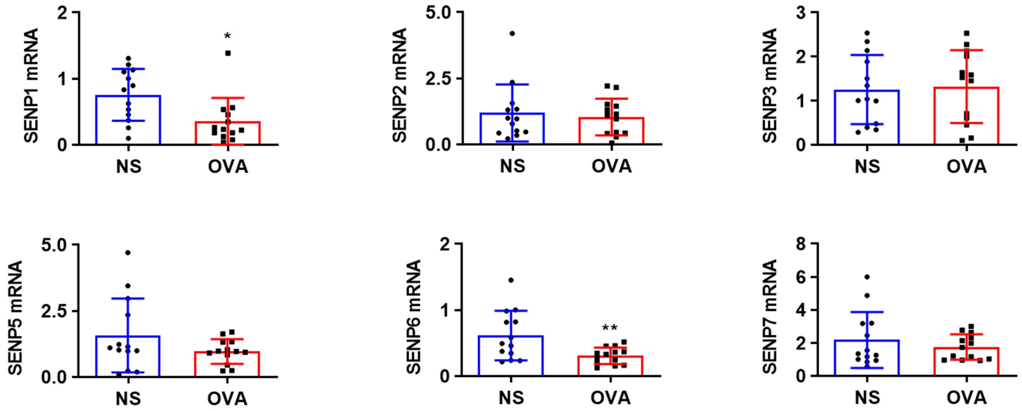

Supplementary Fig. 1 DeSUMOylation enzymes are downregulated in allergic airway epithelia. OVA-sensitized mice were aerosolized with 1% OVA or an equal volume of NS for 30 min, once daily for 7 d. Lungs were subjected to RNA isolation and quantitative RT-PCR (*P* values: 0.021, 0.0085). Mean ± SD, n=13, unpaired two-tailed Student's *t*-test, **P*<0.05, ***P*<0.01. Source data are provided as a Source Data file.

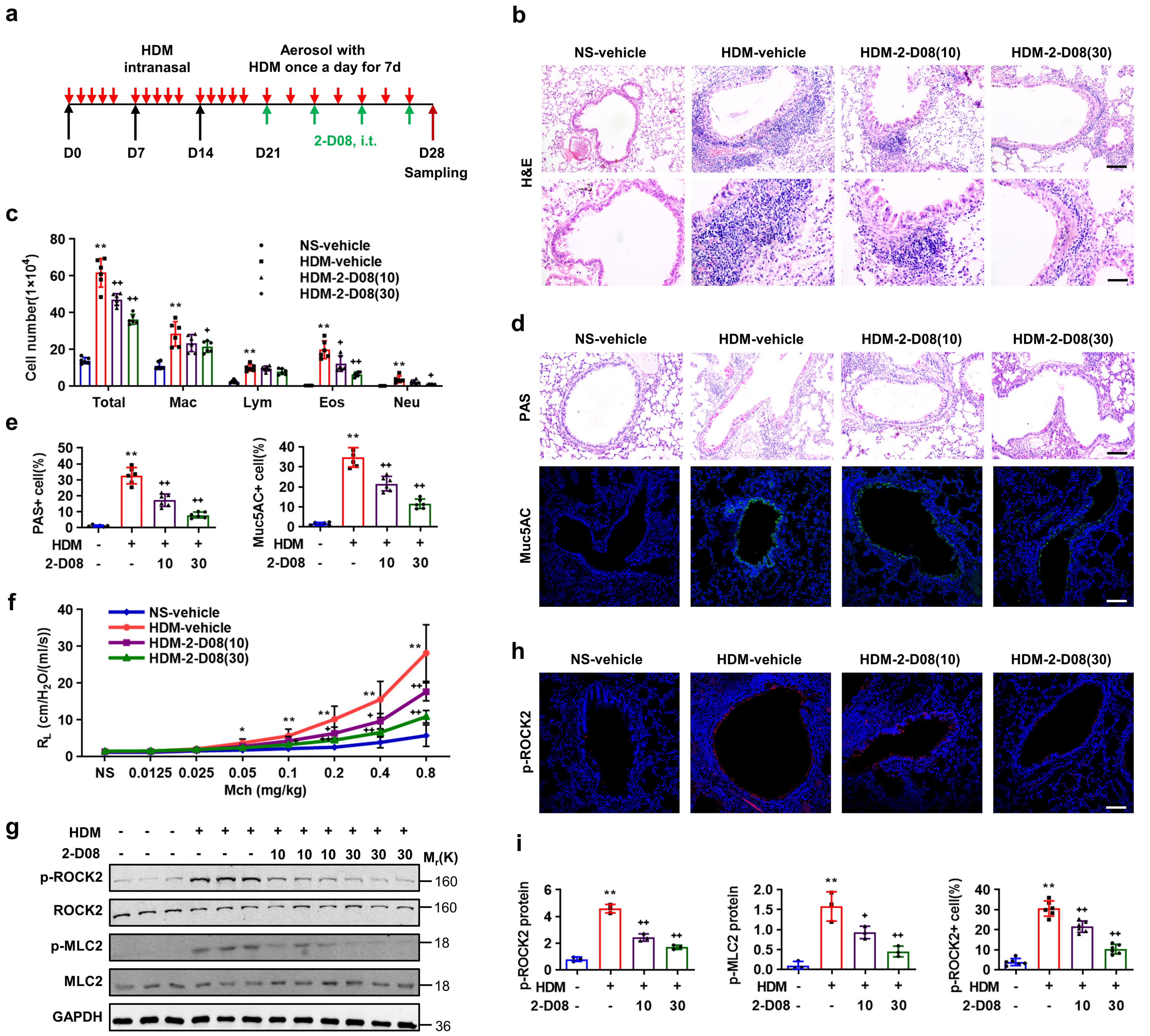

Supplementary Fig. 2 Suppression of SUMOylation attenuates HDM-induced goblet cell metaplasia and inactivates ROCK2. a From day 21 to 27, HDM-sensitized mice were aerosolized with aerosolized with 0.5‰ HDM or an equal volume of normal saline for 30 min to trigger recall responses. From day 21, 50 µl/mouse of 2-D08 at the concentration of 10 and 30 mg/kg or vehicle was intratracheally administered at 2 h post each HDM aerosolization, every other day. On day 28, mice were subjected to a variety of analyses and euthanized for sampling (each n=8). **b**-**f** 24 h after the last HDM challenge, BALFs were prepared for cell counting and classification (**c**, *P* values: <0.0001, 0.0001, <0.0001, <0.0001, 0.0009; 0.0019, 0.0161; <0.0001, 0.0416, <0.0001, 0.0112), lungs were subjected to paraffin-embedded sectioning for H&E staining (**b**, scale bar, 10 µm or 5µm), PAS staining and immunostaining for Muc5AC (**d,** scale bar, 10 µm) and examination of methacholine-provoked airway hyperreactivity (**f**, *P* values: 0.0136, 0.0015, 0.0005, 0.0003, <0.0001; 0.0394, 0.023, 0.0092; 0.0107, 0.0034, 0.0015, 0.0003), semi-quantification of PAS and Muc5AC (**e**, *P* values: <0.0001, 0.0002, <0.0001; <0.0001, 0.0003, <0.0001). **g**-**i** Lungs and bronchus were subjected to immunofluorescence staining and western analyses, respectively (each n=6 or 3, *P* values: <0.0001, 0.0009, 0.0001; 0.0026, 0.047, 0.0074; <0.0001, 0.0009, <0.0001). Scale bar, 10 µm. Mean ± SD, One-way ANOVA and Tukey-Kramer multiple comparisons test, *,$^{+}$*P*<0.05, **,$^{++}$*P*<0.01. Source data are provided as a Source Data file.

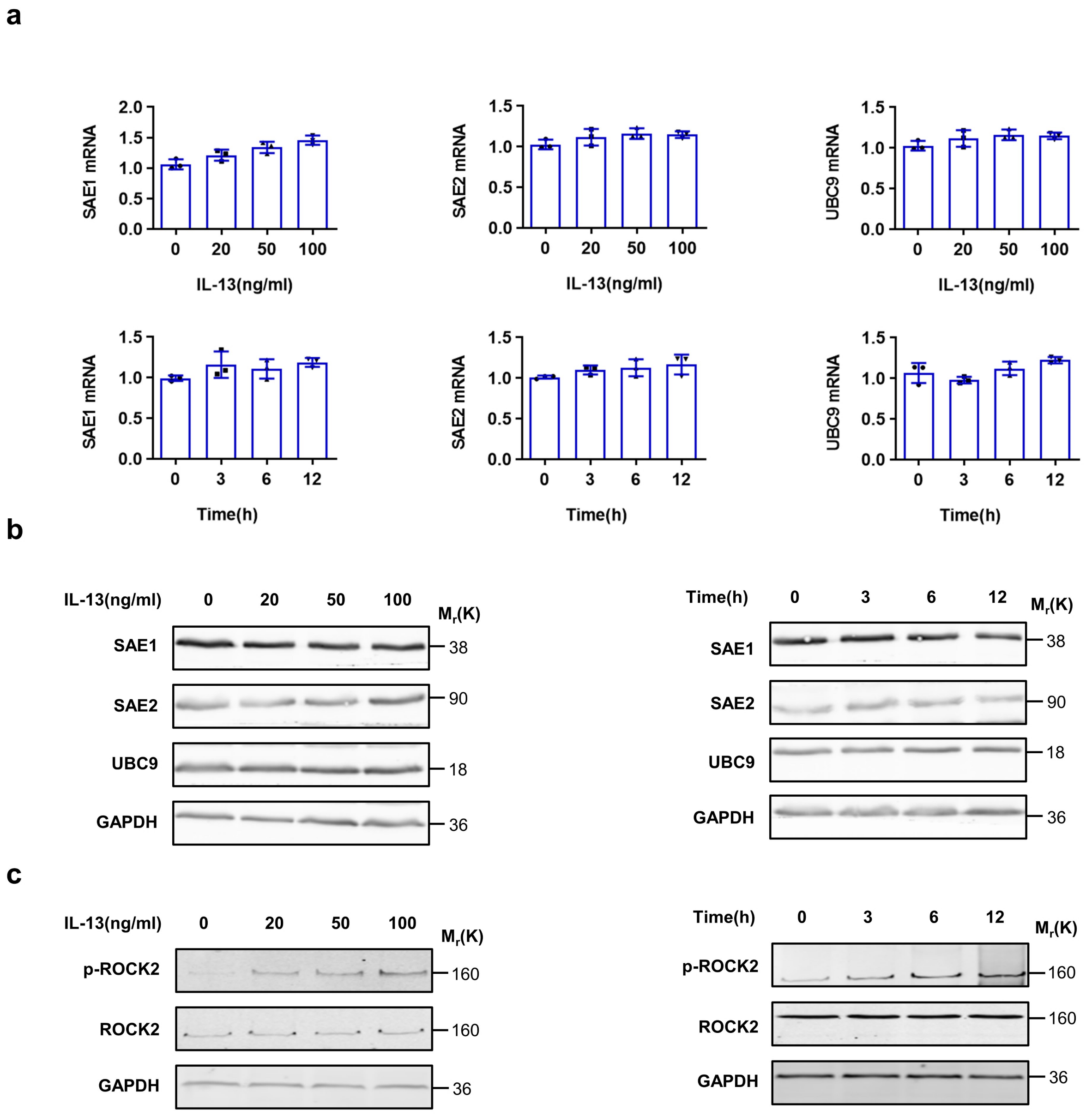

Supplementary Fig. 3 IL-13 induces the phosphorylation of ROCK2 in 16HBE cells. a, **b** Quantitative RT-PCR (n=3) and western analyses of SAE, SAE2 and UBC9 after IL-13 treatment at the indicated concentrations in 16HBE cells for 12 h or at 100 ng/ml for the indicated times. **c** Western analyses of p-ROCK2 and ROCK2 after IL-13 at the indicated concentrations in 16HBE cells for 12 h or at 100 ng/ml for the indicated times. Mean ± SD, One-way ANOVA and Tukey-Kramer multiple comparisons test. Experiments were repeated independently at least three times with similar results. Source data are provided as a Source Data file.

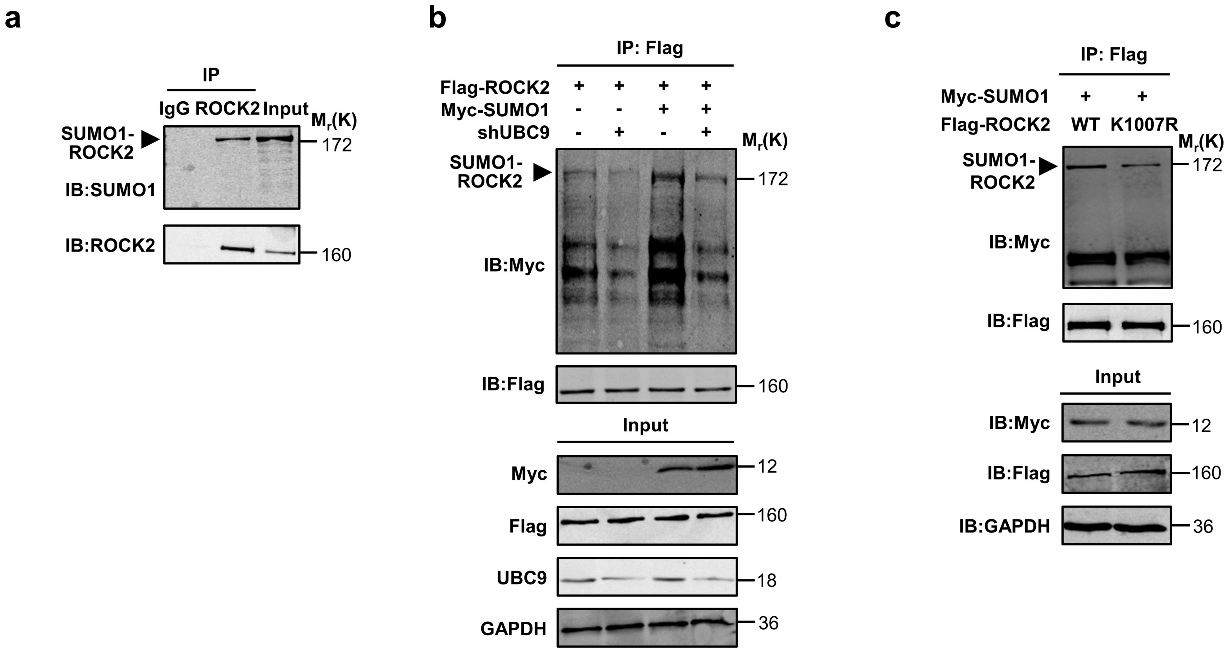

Supplementary Fig. 4 SUMOylation on K1007 activates ROCK2 in 293T cells. a Co-immunoprecipitation experiments using a control IgG or a ROCK2 antibody in 293T cells. **b** Co-immunoprecipitation experiments using a Flag antibody in 293T cells transfected with Flag-ROCK2 in combination with Myc-SUMO1 in the presence of scramble or Ubc9 shRNA. **c** Co-immunoprecipitation experiments using a Flag antibody in 293T cells transfected with Myc-SUMO1 and Flag-ROCK2/Flag-ROCK2(K1007R). Experiments were repeated independently at least three times with similar results. Source data are provided as a Source Data file.

Position	Peptide	score	Cutoff	P-value
88	KIRGLQMKAEDYDVV	12.49	3.24	0.009
216	GLIHRDVKPDNMLLD	9.696	3.24	0.013
238	ADFGTCMKMDETGMV	7.404	3.24	0.026
355	IRQHPFFKNDQWHWD	3.508	3.24	0.034
615	LLETAKLKLEKEFIN	9.372	3.24	0.011
795	DVRNLTLKIEQETQK	14.477	3.24	0.005
828	KMSEKQLKQENNHLM	17.65	3.24	0.003
884	KTQVRELKEECEEKT	10.819	3.24	0.007
1007	QEQLSRLKDEEISAA	11.502	3.24	0.007
1049	HKDHMDKKEEIIAPC	5.163	3.24	0.012
1071	RKLHMELKSEREKLT	9.446	3.24	0.047

Supplementary Fig. 5 Bioinformatics prediction of the hROCK2 sequence revealed several conserved SUMOylation consensus motifs including K88, K216, K238, K355, K615, K795, K828, K884, K1007, K1049, and K1071.

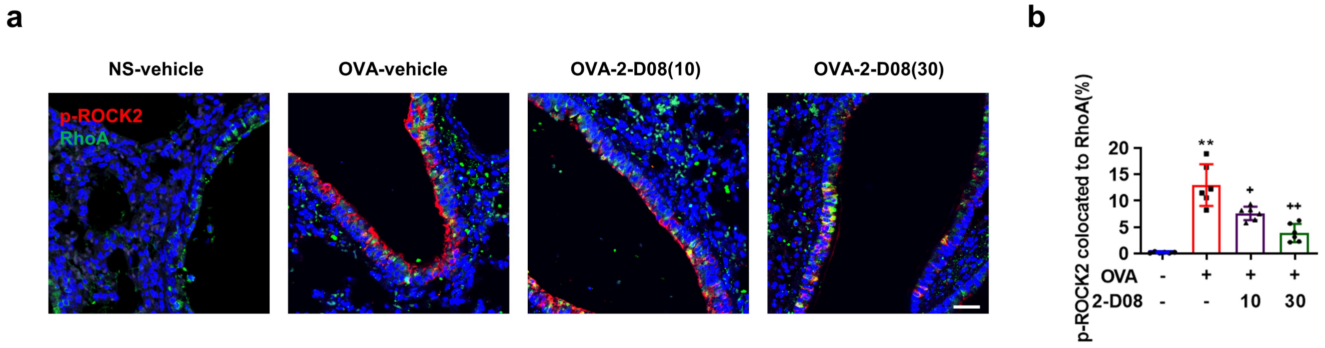

Supplementary Fig. 6 2-D08 treatment affects the co-localization of p-ROCK2 and RhoA in mice lung epithelial cells. a, b co-localization of p-ROCK2 and RhoA in bronchial epithelia of mice challenged with allergen by immunostaining and semi-quantification (*P* values: <0.0001, 0.0102, 0.0004). Scale bar, 5 μm. Mean ± SD, n=6, One-way ANOVA and Tukey-Kramer multiple comparisons test, $^{+}P<0.05$, $^{**,++}P<0.01$. Source data are provided as a Source Data file.

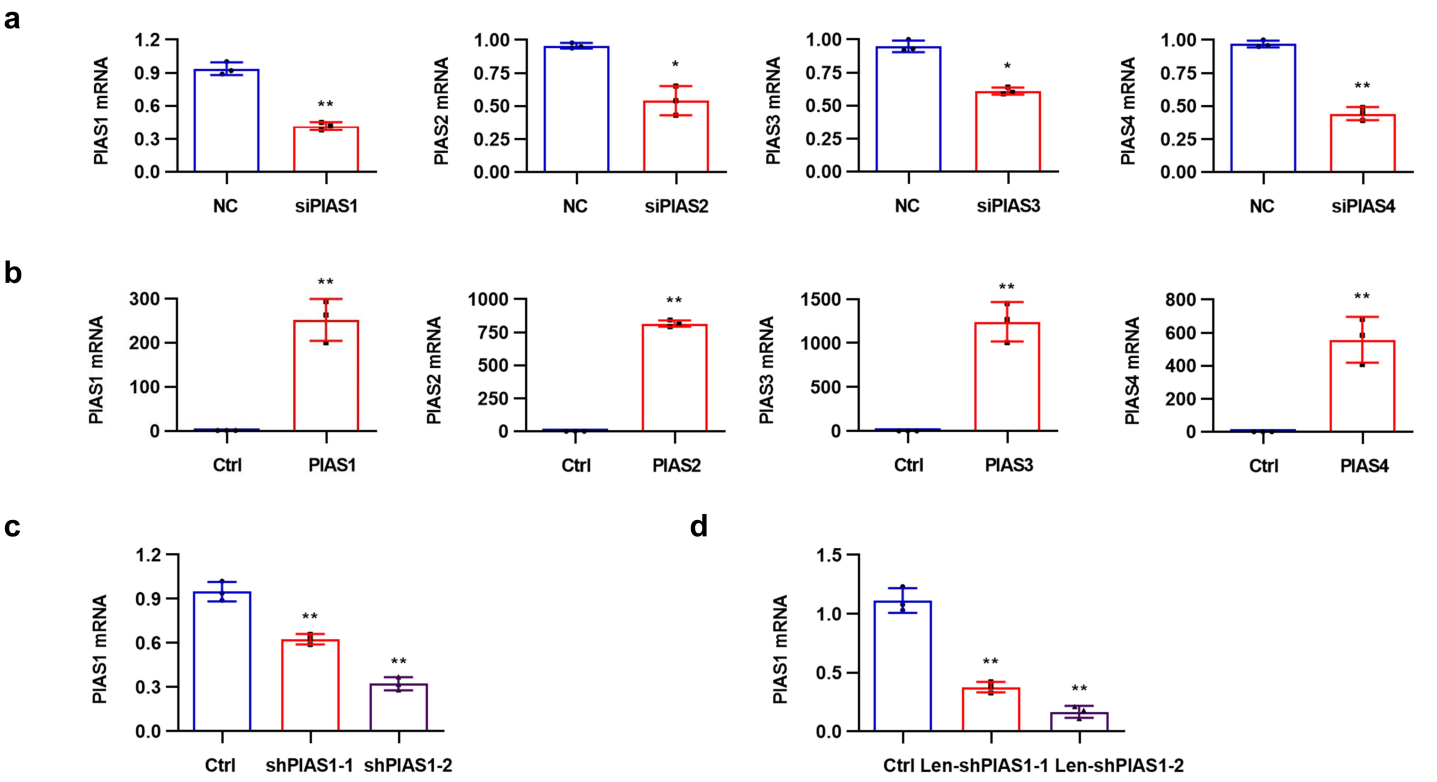

Supplementary Fig. 7 Analysis of the mRNA levels of PIAS1-4 by knockdown and overexpression. a, **b** Quantitative RT-PCR analysis for PIAS1-4 mRNA levels in 293T cells transfected with PIAS1-4 siRNAs or scramble siRNA (NC) or with PIAS1-4 constructs or vector (*P* values: 0.0002, 0.0117, 0.0117, <0.0001; 0.0008, <0.0001, 0.0007, 0.0023). **c**, **d** Quantitative RT-PCR analysis for PIAS1 mRNA levels in 293T cells after infection with lentiviral PIAS1-shRNA or Scramble-shRNA for 48 h (*P* values: 0.0011, 0.0001; 0.0006, 0.0002). Mean ± SD, n=4, unpaired two-tailed Student's *t* test or One-way ANOVA and Tukey-Kramer multiple comparisons test, **P*<0.05, ***P*<0.01. Source data are provided as a Source Data file.

Supplementary table 1： Primer-sequence for qPCR analysis

Gene	Forward primer	Reverse primer
SAE1 (human)	CAGTATGACCGACAGATCCGC	GGAGATACCTGTTCGTGGTCC
SAE2 (human)	CCACATCGACCTGATTGATCTG	GGCAACCTGAGCCTTTGATCT
UBC9 (human)	GGAGGAAGGACCACCCTTTG	GGATAGCGCACTCCCAGTT
PIAS1 (human)	ACAGTGCGGAACTAAAGCAAA	GGACTTGAATGTACGTTGGGG
PIAS1 (mouse)	ACGCAAACACGAACTTCTTACA	TCCGCAGGCGTCATAATTTTC
PIAS2 (human)	GGCTTTGCTGGACGGAATAAA	CGGCGTCTATACAATTCTCGGAT
PIAS3 (human)	CTGGGCGAATTAAAGCACATGG	AAAGCGTCGTCGGTAAAGCTC
PIAS4 (human)	GTGGGCCGGAGTAAGAGTG	TCAGGGCTACAGTCAAACTGC
SENP1 (mouse)	GATTGCGCCGGATTGAAGAG	CATCCTGGTTACCGTTACGAAA
SENP2 (mouse)	AAGAACAGTCTCTACAATGCTGC	CCGATTTCAGCGTAAAACCAAAG
SENP3 (mouse)	CCGGCCATCTTTTGATGCCT	GCGAGGTGCTTTTTGAGTAGAG
SENP5 (mouse)	CCCCAAAACTTGTGCTTTCTGA	AGTAGCCAGTCCAGACTTTGT
SENP6 (mouse)	GTTGTTTGTTTCCCTGGTTTGG	GCACTCGAATCAGTCACAGCTA
SENP7 (mouse)	CTATGGACGGACTTAGGACGA	CAGATGTCGAAGGCAATGAGT
GAPDH (human)	GGAGCGAGATCCCTCCAAAAT	GGCTGTTGTCATACTTCTCATGG
GAPDH (mouse)	TGGCCTTCCGTGTTCCTAC	GAGTTGCTGTTGAAGTCGCA
