# Supplementary material for: SUMOylation of Rho-associated protein kinase 2 induces goblet cell metaplasia in allergic airways
Source: Nat Commun. 2023 Jul 1;14:3887. doi: 10.1038/s41467-023-39600-4 (PMC10314948; doi:10.1038/s41467-023-39600-4)
Supplement: Supplementary file 3 — Description of Additional Supplementary Files [file 41467_2023_39600_MOESM3_ESM.pdf]

Description of Additional Supplementary Files

Title: Supplementary Data 1

Description: To investigate the potential SUMOylation substrates controlling allergic airway goblet cell metaplasia, we performed an unbiased quantitative phosphoproteomics analysis in 16HBE cells treated with or without IL-13 and 2-D08. Supplemental data 1 showed that a total of 56211 phosphorylation sites for 5283 phospho-proteins were identified and quantified, and proteins with a fold change greater than or equal to 2.0 and less than or equal to 0.5 with $P<0.05$ were considered significantly differential expression.

Title: Supplementary Data 2

Description: Supplemental data 2 analyzed the protein domain of differentially expressed modified peptide segment in IL-13+2D08 vs PBS+IL-13 group. Domain analysis highlighted both Rho guanine nucleotide exchange factors (RhoGEF) and GTPase-activating proteins (RhoGAP) were most tightly involved in the 2-D08 negating IL-13-induced phosphorylation of target proteins.
