# Supplementary material for: SUMOylation of Rho-associated protein kinase 2 induces goblet cell metaplasia in allergic airways
Source: Nat Commun. 2023 Jul 1;14:3887. doi: 10.1038/s41467-023-39600-4 (PMC10314948; doi:10.1038/s41467-023-39600-4)
Supplement: Supplementary file 6 — Reporting Summary [file 41467_2023_39600_MOESM6_ESM.pdf]

nature portfolio

Corresponding author(s): Prof. Ximei Wu

Last updated by author(s): Jun 12, 2023

Reporting Summary

Nature Portfolio wishes to improve the reproducibility of the work that we publish. This form provides structure for consistency and transparency in reporting. For further information on Nature Portfolio policies, see our Editorial Policies and the Editorial Policy Checklist.

Statistics

For all statistical analyses, confirm that the following items are present in the figure legend, table legend, main text, or Methods section.

n/a	Confirmed	
[ ]	[x]	The exact sample size (*n*) for each experimental group/condition, given as a discrete number and unit of measurement
[ ]	[x]	A statement on whether measurements were taken from distinct samples or whether the same sample was measured repeatedly
[ ]	[x]	The statistical test(s) used AND whether they are one- or two-sided<br>*Only common tests should be described solely by name; describe more complex techniques in the Methods section.*
[x]	[ ]	A description of all covariates tested
[x]	[ ]	A description of any assumptions or corrections, such as tests of normality and adjustment for multiple comparisons
[ ]	[x]	A full description of the statistical parameters including central tendency (e.g. means) or other basic estimates (e.g. regression coefficient) AND variation (e.g. standard deviation) or associated estimates of uncertainty (e.g. confidence intervals)
[ ]	[x]	For null hypothesis testing, the test statistic (e.g. *F*, *t*, *r*) with confidence intervals, effect sizes, degrees of freedom and *P* value noted<br>*Give P values as exact values whenever suitable.*
[x]	[ ]	For Bayesian analysis, information on the choice of priors and Markov chain Monte Carlo settings
[x]	[ ]	For hierarchical and complex designs, identification of the appropriate level for tests and full reporting of outcomes
[x]	[ ]	Estimates of effect sizes (e.g. Cohen's *d*, Pearson's *r*), indicating how they were calculated

Our web collection on statistics for biologists contains articles on many of the points above.

Software and code

Policy information about availability of computer code

Data collection	Samples were captured by Olympus confocal fluorescence microscope. Histomorphometry analyses for PAS, immunohistochemistry and immunofluorescence staining were performed by using Image-Pro Plus 6.0 software (Media Cybernetics, Silver Spring, MD). Airway responsiveness was assessed by the AniRes2005 software.
Data analysis	Numerical data are expressed as means ± SD. Statistics was performed by using Student's t test or One-way ANOVA and Tukey-Kramer multiple comparisons test (SPSS 26.0, SPSS Inc., Chicago, IL). Statistical significance was assessed at levels of P<0.05 and P<0.01. Experiments were repeated at least three times with similar results, and representative results were shown.

For manuscripts utilizing custom algorithms or software that are central to the research but not yet described in published literature, software must be made available to editors and reviewers. We strongly encourage code deposition in a community repository (e.g. GitHub). See the Nature Portfolio guidelines for submitting code & software for further information.

Data

Policy information about availability of data

All manuscripts must include a data availability statement. This statement should provide the following information, where applicable:

- Accession codes, unique identifiers, or web links for publicly available datasets
- A description of any restrictions on data availability
- For clinical datasets or third party data, please ensure that the statement adheres to our policy

The data that support this study are available within the article and its Supplementary Information files. Source data are provided with this paper.

Human research participants

Policy information about studies involving human research participants and Sex and Gender in Research.

Reporting on sex and gender	Our findings do not apply to just one sex and gender. The patients of healthy lobar bronchi are two males and two females.The patients of FBA are three males and one females, and the patients asthma are four males and three females.
Population characteristics	Bronchoalveolar lavage fluids (BALFs) were from four children (5-8 years of age) discharged with a diagnosis of FBA and seven children (4-8 years of age) discharged with a diagnosis of allergic asthma. Human healthy lobar bronchi were surgically obtained from 4 patients (34-56 year of age), who had peripheral lung cancers and underwent pulmonary lobectomy .
Recruitment	Patients who had peripheral lung cancers and underwent pulmonary lobectomy were recruited from patients who visited Department of Thoracic Surgery, the Affiliated Hospital of Jiaxing University. These patients were diagnosed with lung cancer after a series of tests, no other diseases and a long history of medication.<br>Asthma patients and foreign body aspiration (FBA) patients were recruited from patients who visited the Children's Hospital of Zhejiang University School of Medicine. All patients underwent diagnostic fiberbronchoscopy. Asthmatic children satisfied the American Thoracic Society criteria for asthma, including proven reactivity to skin allergen prick tests; they were characterized using spirometry and current symptom levels. All children with FBA, previously healthy, presented with a history of FBA 6 hours before admission; bronchoscopy found food, bone and rock in the bronchi, and the foreign bodies were successfully extracted.
Ethics oversight	All subjects enrolled in this study provided written informed consent .<br>The usage of bronchial tissues in the present study was approved by the Clinical Research Ethics Committee of the Affiliated Hospital of Jiaxing University (LS2020-171).<br>The present study using the remnant BALFs was approved by the Ethics Committee of the Children's Hospital of Zhejiang University School of Medicine (2015-HP-037).

Note that full information on the approval of the study protocol must also be provided in the manuscript.

Field-specific reporting

Please select the one below that is the best fit for your research. If you are not sure, read the appropriate sections before making your selection.

☒ Life sciences ☐ Behavioural & social sciences ☐ Ecological, evolutionary & environmental sciences

For a reference copy of the document with all sections, see nature.com/documents/nr-reporting-summary-flat.pdf

Life sciences study design

All studies must disclose on these points even when the disclosure is negative.

Sample size	Sample size for each experiment is indicated in the legend. For clinical samples, no sample-size calculation was performed because sample size was not chosen based on statistics and solely on availability of patient specimens. For experiments of cells and animals, Sample size was determined based on privous studies and preliminary experiments.
Data exclusions	Data were not excluded from analysis.
Replication	Experiments were repeated at least three times, and representative results were shown.
Randomization	Randomization was not applicable to the clinical samples study. For in vitro experiments of this study, randomization is not relevant. However, all experimental conditions were carefully controlled to ensure same experiment conditions were used for all groups. For animal experiments, BALB/c mice and mice with the same genotype were randomly assigned to control and experimental groups before the experiment.

Blinding	For histology studies, quantification of staining was determined by an individual unaware of the sample's identity.

Reporting for specific materials, systems and methods

We require information from authors about some types of materials, experimental systems and methods used in many studies. Here, indicate whether each material, system or method listed is relevant to your study. If you are not sure if a list item applies to your research, read the appropriate section before selecting a response.

Materials & experimental systems

n/a	Involved in the study
☐	☒ Antibodies
☐	☒ Eukaryotic cell lines
☒	☐ Palaeontology and archaeology
☐	☒ Animals and other organisms
☒	☐ Clinical data
☒	☐ Dual use research of concern

Methods

n/a	Involved in the study
☒	☐ ChIP-seq
☒	☐ Flow cytometry
☒	☐ MRI-based neuroimaging

Antibodies

Antibodies used	p-ROCK2 (S1366, ab228008, Abcam, Cambridge, UK), ROCK2 (ab228000, Abcam), SAE1 (ab185552, Abcam), SAE2 (ab185955, Abcam), UBC9 (ab75854, Abcam), PIAS1 (ab109388, Abcam), Muc5AC (ab3649, Abcam), RhoA (ab187207, Abcam), CC10 (sc365992, Santa Cruz Biotechnology, Santa Cruz, CA), p-MLC2 (S19, 3671, CST, Danvers, MA), MLC2 (3672, CST), SUMO1 (4930, CST), Flag (14793, CST), Myc (2276, CST), HA (3724, CST), GAPDH (SC-32233, Santa Cruz), IRDye 680 and 800 secondary antibodies (LI-COR Biosciences, Lincoln, NE, 1:10000), Alexa555 or Alexa488-conjugated secondary antibody (Invitrogen, Grand Island, NY, 1:1000).

Validation

p-ROCK2 (S1366, ab228008, Abcam, Cambridge, UK) antibody has been validated by Abcam by demonstrating WB, IHC-P, ICC/IF on rat, mouse and human (see website).
ROCK2 (ab228000, Abcam) antibody has been validated by Abcam by demonstrating WB, IP, IHC-P, ICC/IF on mouse and human (see website).
SAE1 (ab185552, Abcam) antibody has been validated by Abcam by demonstrating Flow Cyt (Intra), WB, ICC/IF, IP on human (see website).
SAE2 (ab185955, Abcam) antibody has been validated by Abcam by demonstrating IHC-P, IP, ICC/IF, WB on rat, mouse and human (see website).
UBC9 (ab75854, Abcam) antibody has been validated by Abcam by demonstrating Flow Cyt (Intra), WB, IP, IHC-P, ICC/IF on human (see website).
PIAS1 (ab109388, Abcam) antibody has been validated by Abcam by demonstrating Flow Cyt, ICC/IF, WB, IHC-P on rat, mouse and human (see website).
Muc5AC (ab3649, Abcam) antibody has been validated by Abcam by demonstrating ICC, IHC-Fr, IHC, IHC-P on rat, mouse and human (see website).
RhoA (ab187207, Abcam) antibody has been validated by Abcam by demonstratingFlow Cyt (Intra), ICC/IF, WB on rat, mouse and human (see website).
p-MLC2 (S19, 3671, CST, Danvers, MA) antibody has been validated by CST by demonstrating WB and IF on rat, mouse, drosophila melanogaster and human (see website).
MLC2 (3672, CST) antibody has been validated by CST by demonstrating WB on rat, mouse, and human (see website).
SUMO1 (4930, CST) antibody has been validated by CST by demonstrating WB, IHC and IF on rat, mouse, monkey and human (see website).
Flag (14793, CST) antibody has been validated by CST by demonstrating WB, IHC, IP, F, ChIP and IF on all species expected (see website).
Myc (2276, CST) antibody has been validated by CST by demonstrating WB, IHC, IP, F, ChIP and IF on all species expected (see website).
HA (3724, CST) antibody has been validated by CST by demonstrating WB, IHC, IP, F, ChIP and IF on all species expected (see website).
CC10 (sc365992, Santa Cruz Biotechnology, Santa Cruz, CA) antibody has been validated by Santa Cruz Biotechnology by demonstrating WB, IHC, IP, Elisa and IF on rat, mouse and human (see website).
GAPDH (SC-32233, Santa Cruz) ntibody has been validated by Santa Cruz Biotechnology by demonstrating WB, IP, and IF on rat, mouse, rabbit and human (see website).
IRDye 680 anti mouse secondary antibodies (LI-COR Biosciences, Lincoln, NE, 1:10000, see website).
RDye 800 anti rabbit secondary antibodies (LI-COR Biosciences, Lincoln, NE, 1:10000, see website).
Alexa555 anti mouse/rabbit conjugated secondary antibody (Invitrogen, Grand Island, NY, 1:1000, see website).
Alexa488 anti mouse/rabbit conjugated secondary antibody (Invitrogen, Grand Island, NY, 1:1000, see website).

Eukaryotic cell lines

Policy information about cell lines and Sex and Gender in Research

Cell line source(s)	HEK293T cells (human, ATCC CRL-1573)

	16HBE cells (human, from Huahao. Shen lab)
Authentication	None of the cell lines have been authenticated.
Mycoplasma contamination	Cell lines were tested for mycoplasma contamination and no indication of contamination was dectected.
Commonly misidentified lines (See ICLAC register)	No commonly misidentified cell lines were used.

Animals and other research organisms

Policy information about studies involving animals; ARRIVE guidelines recommended for reporting animal research, and Sex and Gender in Research

Laboratory animals	C57BL/6J and BALB/c mice at 8 weeks of age were purchased from SLAC Laboratory Animal Co. Ltd. (Shanghai, China). The CC10-CreERT2 mouse strain was purchased from Model Animal Research Center of Nanjing University (Nanjing, China). The conditional caRhoA (caRhoA+/-) knock-in mouse strain was generated by Cyagen Biosciences (Santa Clara, CA) as described previously(ref. 13),and the conditional ROCK2(K1007R/+) knock-in founders were generated by CRISPR/Cas9 at Cyagen Biosciences as described previously(ref. 49). 8-week-old mice with a C57BL/6J genetic background were used in the experiments.
Wild animals	No wild animals were used in this study.
Reporting on sex	Our findings do not apply to just one sex and gender. For genotypic mice, age- and sex-matched mice were used in the experiments.
Field-collected samples	No field-collected samples were used in this study.
Ethics oversight	The animal protocols were approved by the Zhejiang University Institutional Animal Care and Use Committee.

Note that full information on the approval of the study protocol must also be provided in the manuscript.
